# A case report of sinus node–sparing hybrid ablation for refractory sinus tachycardia following cardioneuroablation for sinus node dysfunction

**DOI:** 10.1186/s12872-025-05255-w

**Published:** 2025-11-07

**Authors:** Sebastian Stec, Piotr Suwalski, Mark la Meir, Carlo de Asmundis, Marta Kornaszewska, Mariusz Kowalewski

**Affiliations:** 1 Department of Cardiac Surgery and Transplantology, National Medical Institute of the Ministry of Interior, Warsaw, Poland; 2 Elmedica EP-Network, SKA, Skarzysko-Kamienna, Poland; 3 Cardiac Surgery Department, Universitair Ziekenhuis Brussel-Vrije Universiteit Brussel, Brussels, Belgium; 4 Heart Rhythm Management Centre, Postgraduate Program in Cardiac Electrophysiology and Pacing, Universitair Ziekenhuis Brussel - Vrije Universiteit Brussel, European Reference Networks Guard-Heart, Brussels, Belgium; 5 Thoracic Research Center, Collegium Medicum Nicolaus Copernicus University, Innovative Medical Forum, Bydgoszcz, Poland

**Keywords:** Inappropriate sinus tachycardia, Cardiac ablation, Sinus node, Hybrid ablation, Heart team

## Abstract

**Background:**

Cardioneuroablation (CNA) is increasingly used worldwide in the treatment of functional bradyarrhythmia mediated by excessive vagal tone. However, a potential early or long-term complication is the development of postprocedural inappropriate sinus tachycardia (IST), which remains difficult to manage. Recent data suggest that sinus node (SN)–sparing hybrid ablation may offer promising long-term outcomes in patients with IST and postural orthostatic tachycardia syndrome (POTS). We present what is, to our knowledge, the first documented case of such a procedure performed for IST/POTS following an uncomplicated CNA for symptomatic vagally mediated sinus bradycardia (SB). The comprehensive treatment strategy included on-site cardiac rehabilitation, a home-based telerehabilitation program, and evaluation using cardiovascular autonomic functional testing (CAFT) and the Malmö POTS scoring system.

**Case summary:**

We present a 33-year-old woman with a 6-month history of dizziness, palpitations, exercise and orthostatic intolerance, dyspnea, presyncope, and one syncope episode. Symptoms of IST (130–170 bpm) appeared within 1 week after CNA for symptomatic SB. Despite the diagnosis of IST, CAFT have confirmed POTS. Other causes of sinus tachycardia (ST) were excluded according to guidelines. Nonpharmacological and pharmacological treatment proved ineffective. Following shared decision-making, the patient was referred for SN-sparing hybrid ablation with right-sided video-assisted thoracoscopic surgery (VATS). The patient subsequently participated in hybrid cardiac rehabilitation. At the 3-month follow-up, she was drug free and maintained a normal sinus rhythm. No evidence of bradycardia, IST/POTS, or vasovagal syncope (VVS), including CAFT, was documented during the follow-up. The serial MALMO POTS scoring system before and 3, 6, 9, 12 and 18 months after SN-sparing hybrid ablation demonstrated consistent and significant improvement, with scores decreasing from 46 to 13, 10, 6 and 12 points, respectively, values comparable to those observed in the healthy population.

**Conclusions:**

This is the first reported case of SN-sparing hybrid ablation for IST/POTS that developed after primary, uncomplicated CNA. Although not yet included in guidelines, the implementation of both procedures for cardiovascular autonomic dysfunction (CVAD) requires comprehensive and multidisciplinary heart team management. The MALMO POTS scoring system might be a useful tool for assessing CVAD before and after cardioneuromodulation procedures and further comprehensive evaluation.

## Background

 Cardioneuroablation (CNA) is being implemented worldwide for the treatment of functional vagally mediated bradyarrhythmias [1]. However, although one of its early or long-term severe complication is development of post-procedural symptomatic inappropriate sinus tachycardia (IST) [2–6], this phenomenon is considered rare and underreported, with only isolated cases reported in the literature. No quantitative data on the incidence of persistent IST or postural orthostatic tachycardia syndrome (POTS) after CNA are currently available, as patients with prior CNA have not been included in large studies such as SUSRUTA-IST [7] or HEAL-IST [8], and our case appears to be among the first described. A similar observation was recently reported by Kulakowski and Piotrowski [9], who highlighted this underappreciated and underreported adverse effect. The management of both spontaneous and post-CNA IST remains challenging. However, recent studies suggest sinus node (SN)-sparing hybrid ablation is associated with promising long-term results in patients with IST and POTS [7, 8, 10, 11]. Hybrid ablation referred to single stage procedure with simultaneous presence of electrophysiologist and cardiac surgeon during entire procedure of SNS and three dimensional zero-fluoroscopy endocardial mapping and epicardial access for video assisted thoracoscopic surgery. In some patients additional touch-up endocardial applications and catheter ablation is required during the same SNS procedure.

In the present case, the occurrence of IST/POTS after CNA is considered more likely to reflect the unmasking of an underlying arrhythmogenic substrate—specifically a reentry loop involving the superior vena cava sleeve and the crista terminalis—rather than a direct complication of the initial procedure, as supported by recent anatomical mapping studies of the sinus node region (Eltsov et al., 2025) [12] Both, functional bradyarrhythmia’s and IST/POTS may represent signs of persistent and severe cardiovascular autonomic dysfunction (CVAD) associated or not with post-COVID − 19 syndrome, that may require dedicated questionnaires to validate severity of symptoms and treatment progress [13]. 

To our knowledge, we report the first documented case of SN-sparing hybrid ablation for IST/POTS following primary, uncomplicated CNA for symptomatic functional vagally mediated sinus bradycardia. Moreover, our hybrid approach included inpatient cardiac rehabilitation and remote telerehabilitation program and use of cardiovascular autonomic functional tests (CAFT) and MALMO POTS scoring system to evaluate the efficacy of the management. The Malmö POTS scale [13] is a scoring system evaluating 12 distinct symptoms on a scale from 0 to 10, yielding a total score of 0–120 points. A score of 42 points is considered the diagnostic threshold. Since the minimal clinically important change for this scale has not yet been established, in this case improvement was defined as a persistent reduction of the score below the diagnostic threshold and range typical for healthy control group.

## Case presentation

We present the case of a 33-year-old, physically active woman who was referred with a six-month history of dizziness, fatigue, palpitations, exercise and orthostatic intolerance, dyspnoea, pre syncope, and one episode of syncope. These symptoms appeared within the second week after CNA was performed at another institution. Her past medical history included endometriosis. She had a seven-year-old daughter, and her primary goal for treatment was to have the opportunity to become pregnant within one year and return to dancing and trekking.

Before CNA, the patient had a more than 12-month history of progressing symptomatic resting SB with dominant chronic fatigue syndrome, limb coldness, impaired cognitive function and pre syncope only. Although her ambulatory 24-hour Holter monitoring revealed normal values of sinus rhythm (min: 47, mean: 77, max: 142 bpm), several additional on-demand ECG recordings captured symptomatic SB episodes ranging from 20 to 40 bpm. Before CNA, atropine challenge increased the sinus rhythm from 71 to 142 bpm (a 100% increase). CNA was carried out using a step-by-step biatrial approach targeting major ganglionated plexi, mapped according to the modified Pachon’s anatomical method, under 3D electroanatomic guidance and intracardiac echocardiography [1]. Extracardiac vagal nerve stimulation (ECVS) was performed via ultrasound-guided right internal jugular venous access. A quadripolar irrigated catheter was advanced to the level of the jugular foramen; pulsed electric field trains were delivered (1 V/kg, capped at 70 V; 50 µs pulse width; 50 Hz; 5 s) during spontaneous sinus rhythm and proximal coronary-sinus pacing with continuous surface ECG in accordance with the published methodology [14]. A positive vagal response was predefined as ≥ 50% reduction of sinus rate, a sinus pause ≥ 2 s, and/or transient atrioventricular (AV) nodal block. A final ECVS was repeated 15 min after the last RF application to document the degree of parasympathetic denervation. (complete functional denervation). CNA was completed with increase in the sinus heart rate from 70 to only 85 bpm and the disappearance of ECVS-induced sinus arrest and AV block. Moreover, immediately after CNA, no significant changes in sinus rhythm or AV conduction after atropine challenge were noted [10].

Within one week after CNA, symptoms of IST appeared, accompanied by a significant decrease in quality of life. Cardiac ECHO, brain computed tomography and magnetic resonance imaging, and routine biochemistry exams were normal. On 24-hour ECG Holter monitoring (min 70, mean 102, max 170 bpm), several symptoms were correlated with sinus tachykardia (episodes >130 bpm) at rest or with minimal stress. Moreover, prolonged ECG monitoring with an external ECG loop recorder revealed an exaggerated heart rate response exceeding 130–170 bpm, with a normal P wave morphology and axis changing during waking hours, despite ivabradine treatment (2 × 7.5 mg). Other etiologies of ST were excluded through comprehensive interdisciplinary evaluations. According to the guidelines [6], several nonpharmacological recommendations (including maintaining adequate fluid and salt intake, sleep hygiene, exercise, and compression stockings) and pharmacological treatments (bisoprolol and ivabradine at the highest tolerated doses) have failed to alleviate patients’ ST and severe, disabling symptoms (MALMO score: 46).

Moreover, the patient reported progressive deconditioning, exercise intolerance and orthostatic intolerance. Finally, the patient wished to become pregnant, which precluded prolonged and ineffective treatment with ivabradine.

Six months after CNA, the patient was referred for battery of CAFT [15], which included active standing test, the Valsalva maneuver, deep breathing test, head-up tilt table test performed according to the Italian protocol, and bilateral carotid sinus massage [16]. Her active standing test revealed an increase in heart rhythm from 75 to 130 bpm within 3 min, with reproducible symptoms and stable low blood pressure of 95/65 mmHg, confirming the diagnosis of POTS. Carotid sinus massage and the Valsalva maneuver confirmed normal autonomic function; however, the deep breathing test was abnormal, with a difference in sinus rhythm of less than 4 bpm. A head-up tilt test with beat-to-beat blood pressure monitoring (Finapres Nova, Enschede, the Netherlands) revealed borderline criteria for POTS (an increase in sinus rhythm from 75 to 100 bpm within 10 min of testing without hypotension), despite the patient having been off medications for only two days. Three minutes after the administration of 400 µg of sublingual nitroglycerin spray, a vasodepressive vasovagal syncope response was confirmed (sudden drop in blood pressure from 120/90 to 60/20 mmHg with a drop in ST from 125 to 100 bpm and a 2:1 AV block). Atropine challenge at 0.04 mg/kg resulted in only a minimal increase in sinus rhythm (from 80 to 83 bpm) and a slight decrease in the PR interval (from 200 ms to 180 ms).

Than next, on electrophysiological study (EPS), the diagnosis of IST was confirmed by exclusion of any other supraventricular arrhythmias. No ventricular-atrial conduction was observed at baseline or after isoproterenol or atropine infusion. The criteria for complete vagal denervation of the sinus node (SN) were met by ECVS. However, a slight improvement in AV conduction, particularly the Wenckebach point, was observed after the administration of atropine.

We discussed three treatment options with the patient: continuation of pharmacological therapy; conventional endocardial SN modification/ablation; and SN-sparing hybrid ablation. Given the persistent, drug-refractory symptoms and her planned pregnancy, we considered that further pharmacotherapy had a low likelihood of success. Conventional endocardial SN modification was discussed but carries a substantial risk of SN injury and potential pacemaker dependence, which she wished to avoid. However, our heart-team experience with SN-sparing procedures shows favorable outcomes with a low complication rate [7, 17]. We also discussed thoracoscopic, procedure-specific risks (pericardial/pleural complications, pain). The patient prioritized symptom relief while avoiding a pacemaker. Following shared decision-making, the patient was referred for SN-sparing hybrid ablation via video assisted thoracoscopic surgery (VATS). Ivabradine was discontinued prior to surgery.

**Fig. 1:**
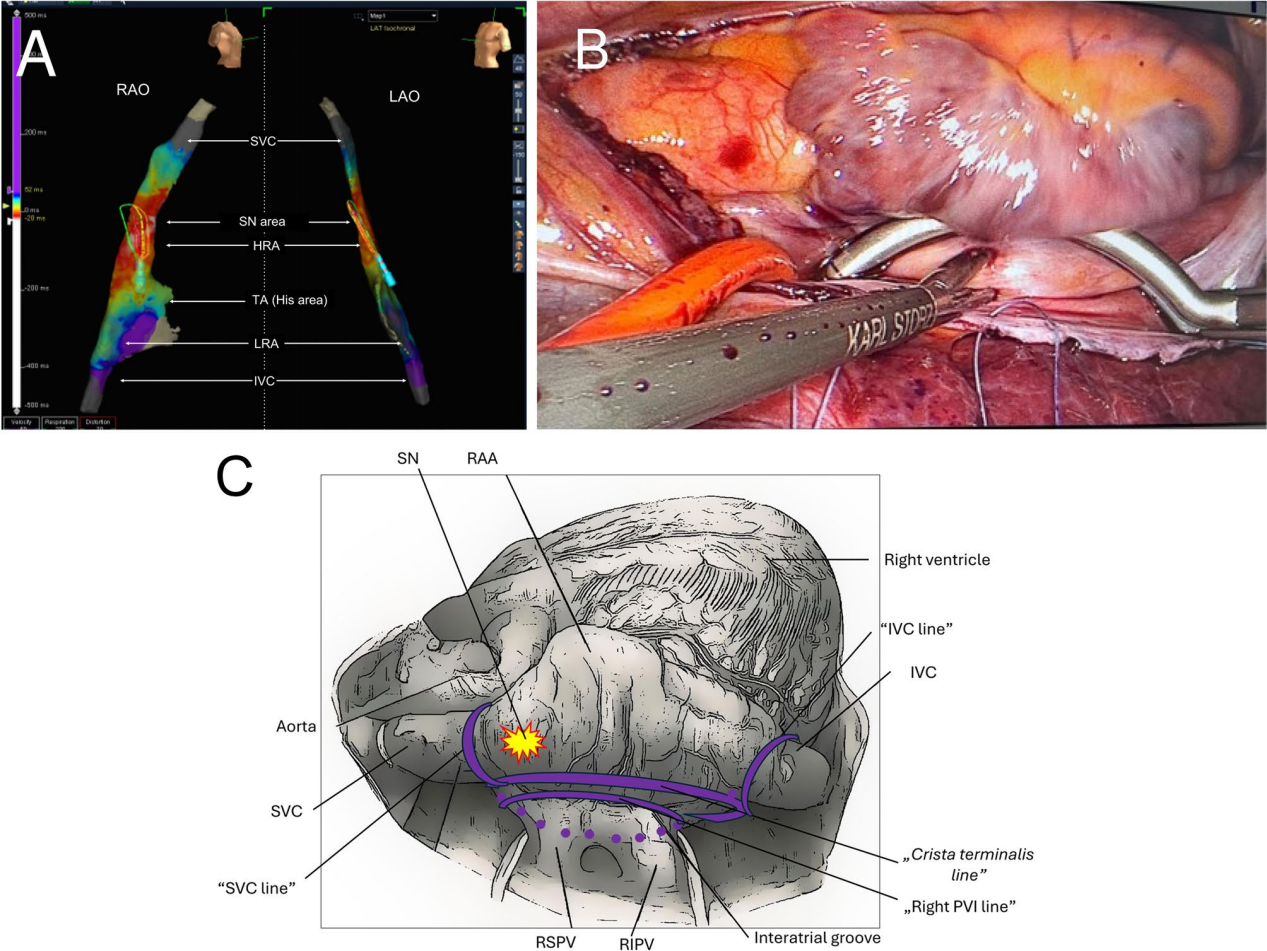
**A** A baseline biplane high-density three-dimensional electrocanatomical endocardial activation map (Advisor HD Grid catheter and Ensite Precision, Abbott, St Paul, MN, USA) of right atrium [superior vena cava (SVC), high right atrium (HRA), low right atrium (LRA), tricuspid annulus (TA)/His area and inferior vena cava (IVC) in LAO (left panel) and RAO view (right panel)]. It was generated from femoral access to validate the baseline earliest local activation time of the sinoatrial node (SN area) during sinus rhythm of 85 bpm. **B** VATS view of the right atrium after opening the pericardium. Crista terminalis line creation via a bipolar isolator synergistic surgical ablation system (AtriCure, Mason, OH, USA). **C** Right-sided thoracoscopic view of the right atrium showing the sinus node–sparing hybrid lesion set. The illustration depicts the epicardial surface of the right atrium and caval veins from a right-sided thoracoscopic perspective. The sinus node region (yellow star) is first identified by combined endocardial activation mapping and epicardial visualization and is deliberately spared. Purple solid lines indicate the planned bipolar epicardial ablation lines: the SVC line at the right atrium–superior vena cava junction to modify/isolates SVC myocardial sleeves; the IVC line at the right atrium–inferior vena cava junction; and the crista terminalis line connecting both caval lesions along the crista terminalis to limit preferential sinus-node exit pathways. Abbreviations: RAA – right atrial appendage; SVC – superior vena cava; IVC – inferior vena cava; RSPV/RIPV – right superior/inferior pulmonary vein; PVI – pulmonary vein isolation; SN – sinus node

A modified approach was performed without atropine infusion and without additional endocardial applications. Atropine was omitted because the patient’s complete SN denervation from prior CNA, and pre and post procedural ECVS, control CAFT, and control EPS/ECVS (> 3 month after baseline CNA) was documented. Moreover, atropine infusion did not change the sinus rhythm. Standardized intra-procedural autonomic testing was not performed because atropine was administered, which precludes a reliable assessment of vagal reflexes. The absence of a chronotropic response to atropine argues against a vagally mediated mechanism and supports a hypothesis of perinodal conduction and/or coupling abnormalities. Therefore, the decrease in the sinus rate did not start from the point of vagal denervation. Furthermore, SN-sparing hybrid ablation was typically performed with double-lumen bronchial intubation and zero-fluoroscopy endocardial mapping via the femoral vein. Standard right-sided chest access was achieved with three 5-mm working ports: a camera port was placed in the fifth intercostal space at the mid-axillary line. Two additional working ports for instruments were placed along the anterior axillary line in the third and seventh intercostal spaces. First, endocardial mapping and visual assessment ruled out the presence of extensive scarring or adhesions from the previous CNA. The SN location was identified and marked with methylene blue, which was based on the position of the endocardial catheter and the earliest local activation time in the sinus rhythm (Fig. 1A). A bipolar bidirectional radiofrequency clamping device (EMR2, AtriCure Inc., Mason, OH, USA) was positioned over the right pulmonary veins and the superior vena cava (SVC) at the junction with the right atrium to isolate the SVC. The same approach was used to isolate the inferior vena cava. To complete the hybrid IST ablation set, the crista terminalis line was created with the clamp positioned in the oblique sinus and the anterior jaw over Waterston’s groove, covering the crista terminalis (Fig. 1B, C).

**Fig. 2:**
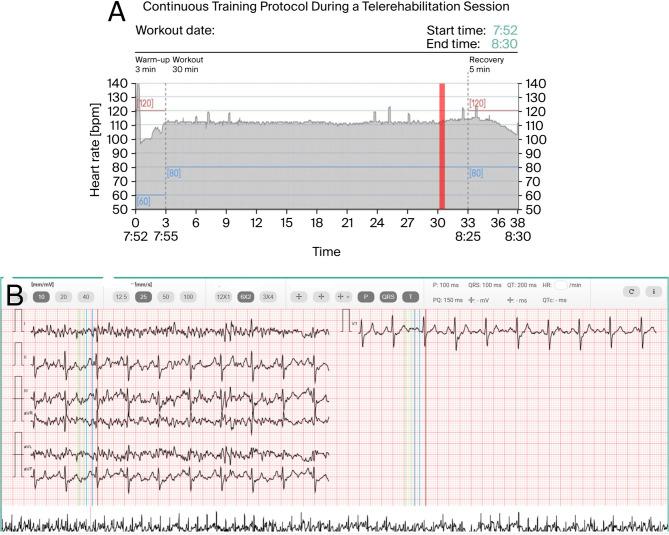
**A** Report from a training session on the 24th day of telerehabilitation, monitored in real time. The training began with a 5-minute warm-up phase, followed by approximately 30 min of exercise at a target heart rate of 80–120 bpm, and concluded with a 5-minute cool-down period. The shaded area represents the recorded heart rate, while the colored lines indicate the predefined limits. **B** Report from the training ECG obtained at the point indicated by the red line in Fig. 2A (27th minute of continuous exercise), showing stable sinus rhythm at 115 bpm. Continuous online monitoring provided high-quality, reliable data, enabling precise patient supervision and confirming good exercise tolerance in this case

After performing typical lines, the patient’s sinus rhythm decreases from 80 to 84 to below 70 bpm, with persistent junctional rhythm. Additional endocardial applications for the crista terminalis or cavo-tricuspid isthmus were omitted to avoid SN/AV node injury during accelerated junctional rhythm persisting after surgical lesions. Then, endocardial mapping confirms the ablation line blocks. The pericardium was closed, and the right lung was inflated. Three days later, the patient was discharged with prophylactic ibuprofen, colchicine, and compression stockings. The patient was subsequently referred for an inpatient cardiac rehabilitation program. Inpatient rehabilitation, conducted according to the FITT principle (Frequency, Intensity, Time, Type) [18], had a comprehensive and multifaceted character. Exercises were performed daily under the individual supervision of specialists, taking into account the patient’s clinical condition and functional capacity. Exercise intensity was gradually increased, starting with breathing and mobilization exercises, progressing to general fitness training, and finally interval endurance training on a cycle ergometer. The program also included psychological support and health education to prevent deconditioning, improve overall fitness, and prepare the patient for a safe transition to the home-based telerehabilitation stage. During a three-week rehabilitation period, the individual reported the disappearance of episodes of IST and symptoms of POTS. The patient participated in a one-month structured telerehabilitation program delivered via a hybrid smartphone platform (DrEryk Kardio, Krakow, Poland). Training sessions, conducted in the form of continuous walking (Nordic Walking), were ECG-monitored in real time by qualified medical staff. All data were automatically transmitted to the platform, and predefined safety thresholds enabled immediate medical intervention if necessary. Throughout the program, the patient demonstrated an appropriate chronotropic response to exercise, reaching a maximum of 140–150 bpm, and within three weeks of observation the junctional rhythm had completely resolved.(Fig. 2A, B).

**Fig. 3:**
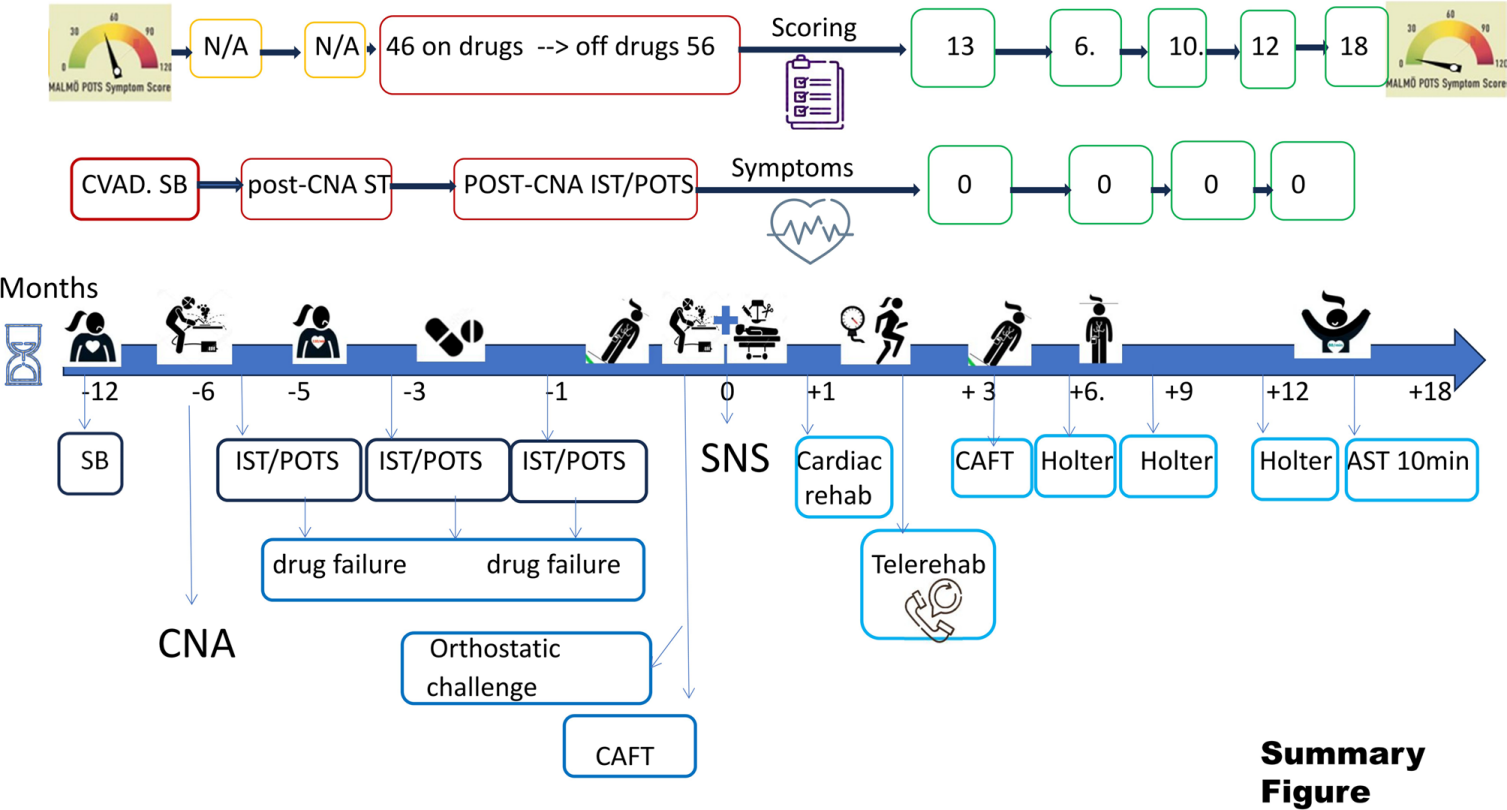
Summary Figure

At the three-month follow-up, the patient was drug free and maintained sinus rhythm within a normal range. Serial 24-hour ECG Holter monitoring between three and twelve months postprocedure demonstrated a mean sinus rhythm below 70 bpm. No symptoms of bradycardia, IST/POTS, or VVS were observed, nor was a significant increase in sinus rhythm after atropine was documented during the control CAFT. She discontinued contraception and agreed to restart jogging, dancing, and trekking, which motivated her invasive treatment. Her MALMO POTS scores at 3, 6, 9, 12, and 18 months after the procedure were 13, 10, 6, and 12 points, respectively (within the range of a healthy population) (Fig. 3).

## Discussion

To our knowledge, this case documented the first SN sparing hybrid ablation for IST/POTS developed after primary uncomplicated CNA for symptomatic vagally-mediated sinus bradycardia. Although significant increase in baseline sinus rhythm is a goal of CNA for symptomatic sinus bradycardia, abnormal increase in specific or unspecific triggers may produce wide range of symptoms of IST/POTS. In a recently published study in patients with reflex asystolic syncope referred for CNA, symptomatic post-procedural persistent or paroxysmal sinus tachycardia was present in 27% of patients and 7% of them required long-term beta-blocker and/or ivabradine therapy up to 18 months [2–5]. Although the occurrence of IST/POTS after CNA is extremely rare, with only isolated cases reported in the literature [2], its manifestation—especially in a persistent or symptomatic form—represents a significant therapeutic challenge. CNA is becoming alternative invasive approach for treatment of functional and vagally mediated bradyarrhythmias.

The etiology of post-CNA IST may share both characteristic and novel mechanisms of typical IST (sympathetic overdrive without local parasympathetic activity, local autoimmunological and inflammatory process, microre-entry induction, genetic and perfusion changes associated with cardioneuroablation and other unknown). Further non-invasive and invasive studies are required to validate the exact mechanisms and SNS efficacy/failure in several subtypes of isolated IST, isolated POTS, IST/POTS and post-CNA IST.

On the other hand, SN sparing procedure is implemented in several centers worldwide and has been superior than SN modification and SN ablation. Moreover, recently SN sparing has beed documented to results in only less than 5% of pacemaker implantation, less than 10% of redo endocardial procedure and no chronic symptomatic phrenic nerve palsy up to 5-year follow-up [7]. Available long-term data indicate more favorable outcomes than typical endocardial SN modification and ablation (with PM implantation) [7]. Moreover, SN sparing is the only accepted invasive procedure for patients with isolated POTS. Therefore, ongoing HEAL-IST trial and registry will provide additional data on efficacy and safety of this procedure. SN sparing is also true hybrid procedure when electrophysiologist and cardiac surgeon support each other and validate the efficacy with combine mapping and ablation. The intent of this SN-sparing hybrid configuration is not to ablate or isolate the sinus node itself, but to modulate perinodal conduction and autonomic inputs. Targeting the SVC/IVC myocardial sleeves and linking them with a crista terminalis line limits preferential sinus node exit pathways, attenuates abnormal drivers, and normalizes SN–atrium coupling without direct SN injury [17].The further complex mechanisms that influence the final result of SNS is currently investigated in HEAL-IST trial and will be validate prospectively by post-procedural autonomic evaluation, 3D mapping and invasive electrophysiologic study including ECVS in the future project OPTIMAL-IST. Some of the failure mechanism is also reported by de Asmundis et al. as far as second procedure is performed [17]. Thanks to minimal invasive VATS and development of new bipolar epicardial ablation system for the achievement of safe the SN sparing may represent a potential area of hybrid approach for treatment of cardiac arrhythmia especially in centers familial with minimal invasive VATS [7, 8, 11, 19]..

Moreover, our case documented the individualized comprehensive approach for cardiac rehabilitation program (inpatient and tele-rehabilitation). It is crucial for the patient safety and reactivation of exercise training with possible early intermittent pericardial effusion, risk of pericarditis, exercise and/or orthostatic intolerance as well as long lasting deconditioning [7, 8, 11].

**Fig. 4:**
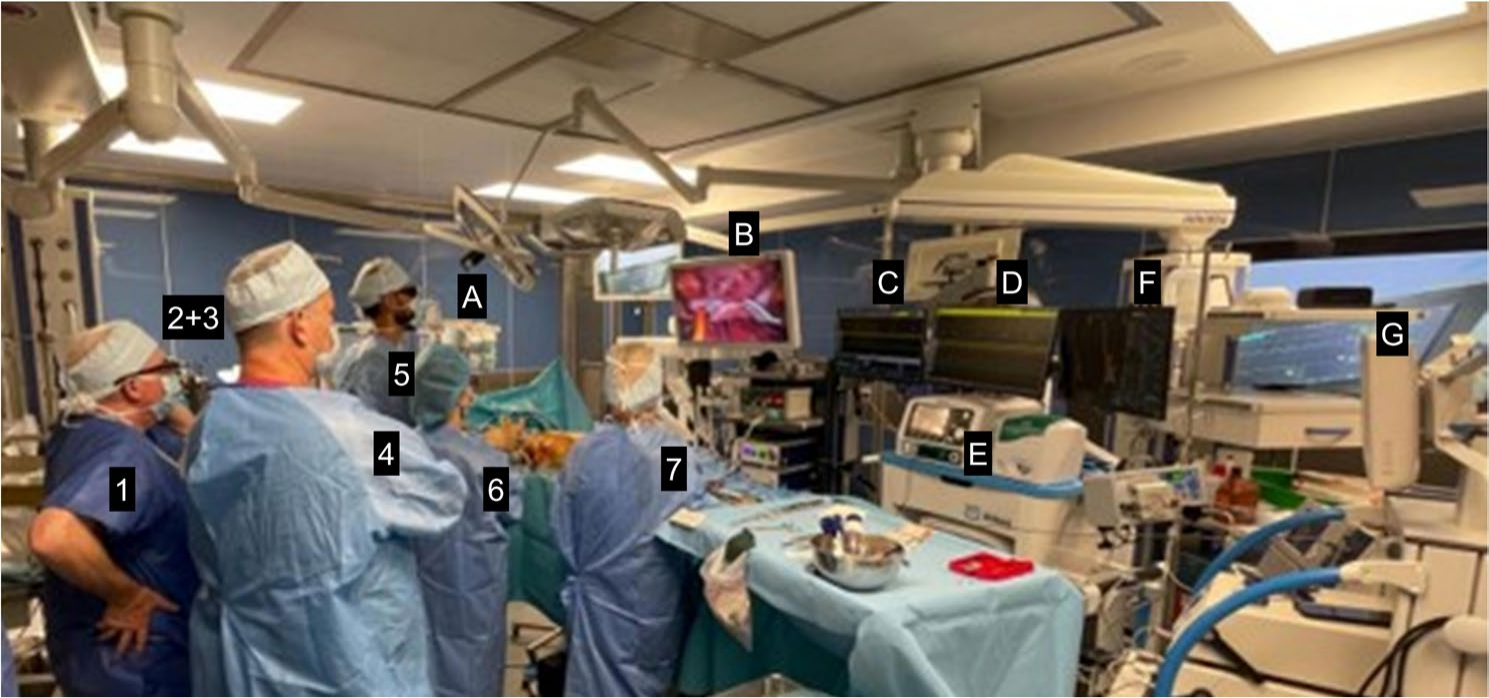
True hybrid procedure equipment and EP-HEART-TEAM "in action": collaboration by the table in EP hybrid operating room during SN-sparing hybrid ablation- from the left EP engineer (1), anesthesiologist and anesthetist nurse (behind others -2+3), electrophysiologists (4), the first operator cardiac surgeons (5), the second operator -cardiac surgeon (6), scrub nurse (7), and perfusionist (not visible) and electroradiologist (not visible). The monitors’ screens (from the left): depth anaesthesia monitor (back to front-**A**), video-assisted thoracoscopic view (**B**), electrophysiological system (extracardiac and intracardiac electrocardiograms - trigger mode) (**C**), electrophysiological system (extracardiac and intracardiac electrocardiograms - review mode) (**D**), RF unipolar generator for endocardial ablation (**E**) , 3D electroanatomical mapping and navigation system (**F**), bipolar RF generator for epicardial ablation (**G**), and backa-up heart-lung machine (not visible) and fluoroscopy machine, (not visible)

As highlighted in the SUSRUTA-IST [7] registry, acute pericarditis represents a potential drawback of the hybrid approach. However, SUSRUTA investigators developed an intensive prophylaxis protocol with high doses of NSAIDs and colchicine, which we have followed from the beginning, and to date we have observed only a single case of transient pericardial effusion (Kowalewski et al., PAIM). Although clinical recovery was observed after the hybrid ablation, it remains uncertain to what extent this improvement resulted from the procedure itself versus the structured cardiac rehabilitation and reversal of deconditioning. It is therefore plausible that the favorable outcome reflected a combined effect of both intervention and rehabilitation. The risks of long-term adverse effects of both CNA and SNS should be monitored and validated before wider implementation of these procedures. In majority of patients not complete parasympathetic denervation or partial re-innervation result in cardioneuromodulation than complete local parasympathetic denervation [19]. This case also required a true multidisciplinary heart team (MDHT) [20] approach, involving physicians (cardiologists, cardiac surgeons, non-invasive cardiologists performing autonomic tests and ECG monitoring, cardiac rehabilitation specialists) as well as allied professionals such as psychologists, psychotherapists, physiotherapists, etc [19–23] (Fig. 4).

Furthermore, the use of the MAPS scale for the assessment of CVAD made it possible to correlate the severity of symptoms with the progression of the disease and the effectiveness of treatment. It is worth noting that the implementation of new techniques, such as CNA, may require the development of novel strategies to prevent adverse effects or complications of this method, particularly in patients with complex CVAD [20–24]. From our perspective, implantation of a loop recorder could represent a valuable tool for long-term monitoring in such patients. However, this option is currently not reimbursed in Poland for other patients than unxplained syncope.

## Conclusions

To our knowledge, this case documented the first case of SN sparing hybrid ablation for IST/POTS developed after primary uncomplicated CNA for symptomatic sinus bradycardia. Although not yet included in current guidelines, implementation of both procedures for patients with CVAD requires comprehensive and MDHT management. Further studies are required to confirm these findings for personalized and participatory approach of patients in the era of interventional neurocardiology.

## Data Availability

All relevant data are included in the article. Further details can be made available upon reasonable request from the corresponding author.

## Abbreviations

AV: Atrioventricular
CAFT: Cardiovascular Autonomic Functional Tests
CNA: Cardioneuroablation
CVAD: Cardiovascular Autonomic Dysfunction
ECG: Electrocardiogram
ECVS: Extracardiac Vagal Nerve Stimulation
EP: Electrophysiology
EPS: Electrophysiological Study
HD: High-density
IST: Inappropriate sinus tachycardia
LAO: Left anterior oblique projection
MDHT: Multidisciplinary Heart Team
POTS: Postural Orthostatic Tachycardia Syndrome
RAO: Right anterior oblique projection
SB: Sinus Bradycardia
SN: Sinus Node
SVC: Superior Vena Cava
VATS: Video-assisted thoracoscopic surgery
VVS: Vasovagal Syncope
